# Evaluating the Nourish Network: a multi-sector collective initiative to progress healthy food retail in Australia

**DOI:** 10.1017/S1368980025100992

**Published:** 2025-09-04

**Authors:** Carmen Vargas, Adyya Gupta, Victoria Hobbs, Anna Peeters, Chris Dubelaar, Christina Zorbas

**Affiliations:** 1 Institute for Health Transformation, Global Centre for Preventive Health and Nutrition, School of Health and Social Development, Faculty of Health, Deakin University, Geelong, Australia; 2 Business School, Faculty of Business and Law, Deakin University, Burwood, Australia

**Keywords:** Evaluation, Collective impact, Healthy food environment, Collective

## Abstract

**Objectives:**

To evaluate the current state of the Nourish Network (NN) – a healthy food retail network, to inform future planning and improvement opportunities.

**Design:**

A qualitative study was conducted using semi-structured interviews conducted between January and April 2024, open-ended survey questions from six online surveys applied between 2019 and 2022 and a focus group with the NN Advisory Committee (NNAC) in June 2024. Thematic analysis was applied to interview and survey data. Results from the thematic analysis were presented to the NNAC, which classified them according to the Strengths, Weaknesses, Opportunities and Threats model, resulting in recommendations for future actions.

**Setting:**

Australia.

**Participants:**

NN members (interviews *n* 9 and survey average response *n* 30) and NNAC (*n* 9).

**Results:**

Nine interviews yielded eight codes clustered into three themes: (i) NN performance, covering overall performance and management since 2018; (ii) members’ engagement with NN activities, addressing current and future involvement and (iii) NN future directions for improvement. The NNAC highlighted strengths in membership diversity and credibility while noting weaknesses in mission clarity and participation. Opportunities for growth include becoming a resource hub through partnerships and national expansion, whereas threats involve limited resources. Recommendations emphasise clear operational tasks, policy alignment and measurement systems to boost accountability and engagement.

**Conclusions:**

To effectively promote healthy food retail changes, the Network for Nutrition and similar organisations must establish a clear vision and enhance stakeholder engagement. This involves consolidating knowledge dissemination, fostering partnerships and securing funding. Ongoing efforts from collectives like the Nourish Network can facilitate research in resource-scarce areas.

Dietary risks are leading contributors to the global burden of disease – accounting for 11 million preventable deaths and 255 disability-adjusted life years in 2017 alone^(1)^. Food retail environments that promote the sale and consumption of foods and beverages are the interface where people make decisions that shape their dietary risks over the long term^(2)^. Despite this, food retail environments are driven by commercial interests, with business-as-usual seldom including considerations about population nutrition and health outcomes^(3)^. However, collective action to address dynamic and complex challenges, such as addressing the global burden of dietary risks, is becoming increasingly important in various fields, including business, healthcare, philanthropy and the military^(4–6)^. One reason for this is that single organisations lack the financial resources, knowledge and legitimacy to act alone on complex issues and respond to widespread public concerns^(6,7)^. As collaborative networks that involve various stakeholders, such as business organisations, governments and civil society, continue to be implemented to create healthier food environments, there is a need to understand and assess their role and impacts^(4,5)^.

The Nourish Network (NN) is an example of a multi-sector collective to improve the demand, availability and access to healthy, sustainable and affordable food retail across sectors in Australia^(8)^. Networks like this are defined as organisations or agencies that come together with a common interest while still maintaining their independence^(4)^. This means that organisations within the NN have different types of relationships and levels of collaboration. For example, organisations within a nutrition network (e.g. NN) can collaborate on a wide range of activities such as advocacy, prevention, clinical work and public health^(9)^. However, not all organisations and agencies are required to participate in every activity due to the broad nature of the network’s initiatives. While the variety of activities may not be a priority of each organisation’s specific goals, networks like the NN are established to promote inclusivity and exposure of the participating organisations and to drive collective action to a wide range of nutrition and public health areas^(4)^.

Assessing the implementation, functionality and impacts of networks is necessary to promote continuous improvement and increase their effectiveness and performance^(7)^. Network effectiveness is not only determined by its capacity to establish internal systems and structures to achieve its goals. It also depends on a network’s ability to engage its members, sustain their engagement, funding models and adapt as needed^(7)^. Previous literature has described various frameworks and tools to evaluate multiple aspects of networks, coalitions and collaborations^(10)^. These models can describe the type of network (e.g. social network analysis^(10)^, collective impact^(11)^ or knowledge networks^(12)^), evaluate the processes^(13,14)^ (e.g. process, impact or outcome evaluation) or assess the functionality and effectiveness of the network (e.g. structure and governance^(6)^, outcomes or impact of the coalition work^(15)^ or functioning and structure^(16)^). Depending on the objectives of interest, it is possible to have a mix of these types of network evaluations. However, evaluations of networks, coalitions and collaboration are enhanced if they remain flexible and manageable to maximise the value of the assessment^(4,7)^.

Despite the growing implementation of collaborative networks in public health, there remains a limited understanding of how such networks function in practice, particularly in the context of food retail environments. While frameworks such as collective impact^(17)^ and network governance^(18)^ provide theoretical guidance, empirical evaluations of real-world networks are scarce, especially those focused on nutrition and food systems. Moreover, existing evaluations often emphasise structural or outcome metrics, with less attention to the lived experiences of members, internal dynamics and adaptive capacity^(7,11)^. This study addresses these gaps by offering a qualitative, member-informed evaluation of the NN, a unique multi-sector initiative in Australia. By exploring how the NN is perceived, governed and sustained, this research contributes to the evidence base on how collaborative networks can be effectively leveraged to promote healthy food environments – an area of increasing policy and practice relevance^(12,19)^. In line with existing evidence and recommendations from the Center for Social Innovation^(20)^, we took an organic approach to evaluating the performance of the NN. This involved querying how well NN’s mission aligned with its vision, adapted to external shifts, executed its function with excellence and sustained its activities^(21)^. We focused on understanding perspectives on these questions in the members’ experiences over time^(20)^. As such, this study aimed to assess the current state of a healthy food retail network – the NN – with a view to informing future planning and improvement opportunities.

## Methods

### Overview of the Nourish Network

The NN, or Healthy Food Environment Futures Network, was established in Victoria, Australia, in 2018 by a multidisciplinary group of researchers, university campus food retail staff, public health practitioners and partners from the food industry and public health sectors. This initiative was designed to foster collaboration among food and nutrition actors from diverse fields, aiming to generate innovative solutions to create health-enabling food environments. Initially, the NN received funding through a Deakin University grant scheme to establish a network focused on enhancing food environments and improving public health outcomes – this funding supported NN activities for a period of two years. Following this period, funding transitioned to a government grant led by the NN Director. This shift in funding sources allowed the NN to sustain its operations for four years and continue its research efforts. Membership (primarily captured through subscription to the internal newsletter) in the NN was free and voluntary, with three active interest groups and three communities of practice. This structure enabled a diverse range of participants to engage and contribute to the network’s objectives without financial barriers, fostering inclusivity and collaboration among members. The NN was led by Professor Anna Peeters, who played a pivotal role in guiding the network’s direction and ensuring its alignment with broader research and public health nutrition goals. Additionally, there was an advisory board that supported the NN’s activities.

### Study design

A qualitative approach was used to evaluate NN members’ experiences and perceived opportunities to strengthen the network in the future^(22)^. Data were collected using multiple sources of evidence: semi-structured one-on-one interviews, open-ended answers from questionnaires delivered to the NN members and a focus group with members of the NN Advisory Committee (NNAC). Reporting of the study design, results and analysis was informed by the Consolidated Criteria for Reporting Qualitative Research Checklist^(23)^.

### Data sources

Interviews: Semi-structured one-on-one, online interviews were chosen to understand members’ perspectives on the NN activities, the participants’ value attributed to those activities and opportunities for future improvement. An interview guide was developed through an iterative and collaborative process within the research team. The initial framework for the questions was informed by: (1) findings from previous NN member surveys (2019–2022), which highlighted key areas of interest and concern among members; (2) principles of organic network evaluation as outlined by the Center for Social Innovation^(20)^, which emphasize adaptability, member perspectives and ecosystem-level insights and (3) theoretical constructs related to network governance, collective impact and stakeholder engagement^(11,17,18)^. Questions were open-ended to promote detailed and reflective responses and to give participants the opportunity to raise new, relevant topics. To ensure clarity and relevance, the draft guide was pilot tested with one NN member. Feedback from this test helped update the wording, flow and focus of the questions. The final version of the guide included prompts to examine both practical experiences and strategic thoughts, ensuring alignment with the study’s social constructionist framework and the aim of gathering diverse views on the NN’s development and impact (see online supplementary material, Supplemental Table S1 for the final interview guide).

### Participants

Interviews were conducted with a convenience sample of members of the NN. From a list of 382 members, we initially invited 40 members to participate in a one-on-one interview via email (detailing the research aims and objectives) and followed up with them three times to encourage participation. The initial group of participants encompassed a diverse range of member types (e.g. roles and organisations) and the coordinator’s assessment of their level of engagement (i.e. presenter, active collaborator and past). Additionally, an open invitation was posted on the NN social networks (i.e. LinkedIn and X) and the NN interest groups and communities of practice collaborating sites (i.e. MS Teams collaboration group). Participants who responded to the open invitation were asked to contact the first author. The interviews were conducted between January and April 2024 by the first author in English and audio recorded via Zoom^(24)^. During interviews, written notes were taken by the interviewer to capture topics requiring further discussion with subsequent participants. All interviews were transcribed using Zoom transcript as a start, deidentified and cross-checked against the audio recordings.

Surveys: On behalf of the NN, one of the authors distributed anonymous online surveys (*n* 6) to all its members from 2019 to 2022 using Qualtrics. Surveys varied depending on the purpose, including questions to help strategic planning for upcoming years (thirteen questions relevant to our analysis), whether the NN’s aligned aims with members’ interests (five questions), and identifying action teams’ satisfaction with activities (eight questions).

Focus group: A focus group with members of the NNAC was conducted to report and present the findings from the interviews and survey analyses, with the aim of developing recommendations for the future sustainment of the NN. The twelve members of the NNAC were invited via email to attend a face-to-face focus group in June 2024. The schedule used in the focus group was developed for a 60-minute session (see online supplementary material, Supplemental Table S2 for the final focus group guide). Open and closed questions were asked to the NNAC, with responses recorded anonymously using an interactive presentation using the *Mentimeter* platform. During the session, written notes from the session discussions were also taken by the lead researcher to capture participants’ insights to support the interpretation of the findings.

### Data analysis

Thematic analysis was conducted using a combination of deductive and inductive approaches, guided by Braun and Clarke’s six-phase framework for qualitative analysis^(22)^. This hybrid approach allowed the research team to explore both predefined areas of interest and emergent insights from the data. The initial coding framework was developed deductively, based on the interview guide and informed by the study’s evaluation objectives. Predefined codes reflected key domains, including network structure, governance, engagement and sustainability, drawing on established frameworks for evaluating collaborative networks and collective impact initiatives. This ensured that the analysis remained aligned with the research questions and theoretical underpinnings of the study. Following the initial deductive coding, the team engaged in inductive analysis to capture unanticipated themes and nuanced participant perspectives. This involved open coding of interview transcripts, allowing themes to emerge directly from the data without being constrained by the initial framework. Codes were iteratively refined and grouped into broader thematic categories through a process of constant comparison across transcripts. One researcher (CV) conducted the initial coding using NVivo software^(25)^, while a second researcher (CZ) reviewed and cross-checked the codes. Findings were discussed and resolved through regular team meetings, which also served as reflexive spaces to consider how the researchers’ disciplinary backgrounds and relationships to the Nourish Network (NN) might influence interpretation. This collaborative process enhanced the credibility and depth of the analysis.

Rather than aiming for data saturation – a concept increasingly critiqued for its ambiguity in qualitative^(26–28)^ – the team adopted the principle of theoretical sufficiency. This approach acknowledges that complete saturation may be neither attainable nor necessary, particularly in exploratory studies^(28,29)^. Instead, the goal was to collect and analyse enough data to meaningfully address the research questions and support the development of robust, well-grounded themes. Theoretical sufficiency was achieved when additional data no longer contributed substantially new insights to the emerging thematic structure, and when the themes were sufficiently rich to inform practical recommendations for the NN.

Open-ended survey responses were analysed using the final coding framework developed from the interview data, ensuring consistency across data sources. Themes and minor themes were then presented to the NNAC, who validated and expanded upon them using a SWOT (Strengths, Weaknesses, Opportunities, Threats) framework. This participatory step further enhanced the trustworthiness of the findings and informed the development of actionable recommendations.

### Researcher team and reflexivity

All members of the research team are affiliated with Deakin University and bring diverse expertise in qualitative research, public health, business and collaborative methodologies. Four listed authors are active members of the NN, which provided them with valuable contextual knowledge and insight into the network’s operations and evolution. To mitigate potential bias and enhance the credibility of the findings, the data collection and initial coding were led by two researchers (CV and CZ) who were independent of the NN and had no prior involvement in its activities.

The team adopted a social constructionist epistemology^(26)^, acknowledging that knowledge is co-constructed through the interactions between researchers and participants. Reflexivity was embedded throughout the research process. Regular team meetings were held to discuss emerging codes and themes, during which researchers critically reflected on how their disciplinary backgrounds, professional roles and prior experiences and roles with the NN might shape their interpretations. These discussions helped ensure that multiple perspectives were considered and that the analysis remained grounded in the data.

Furthermore, the presentation of preliminary findings to the NN Advisory Committee (NNAC) served as a form of member checking and collaborative interpretation. This process allowed for the validation of themes and the incorporation of stakeholder insights into the final analysis. By engaging both internal and external perspectives, the research team sought to strike a balance between insider knowledge and analytical distance, thereby enhancing the trustworthiness and depth of the evaluation.

## Results

### Interviews and surveys

Nine participants who were NN members from academic institutions (56 %, *n* 5), government organisations (22 %, *n* 2) and non-government organisations (22 %, *n* 2) were interviewed (Table 1). Six surveys with an average response of thirty participants were incorporated into the analysis.

**Table 1.** Characteristics of the interview participants

Participant	Sex	Organisation	Role
1	Male	Academic institution	Researcher
2	Female	Non-government organisation	Project officer
3	Female	Academic institution	Researcher
4	Female	Academic institution	Researcher
5	Male	Government organisation	Health promotion
6	Female	Academic institution	Retail manager
7	Female	Academic institution	Food procurement
8	Male	Non-government organisation	Food procurement
9	Female	Government organisation	Consultant

**Figure 1.**
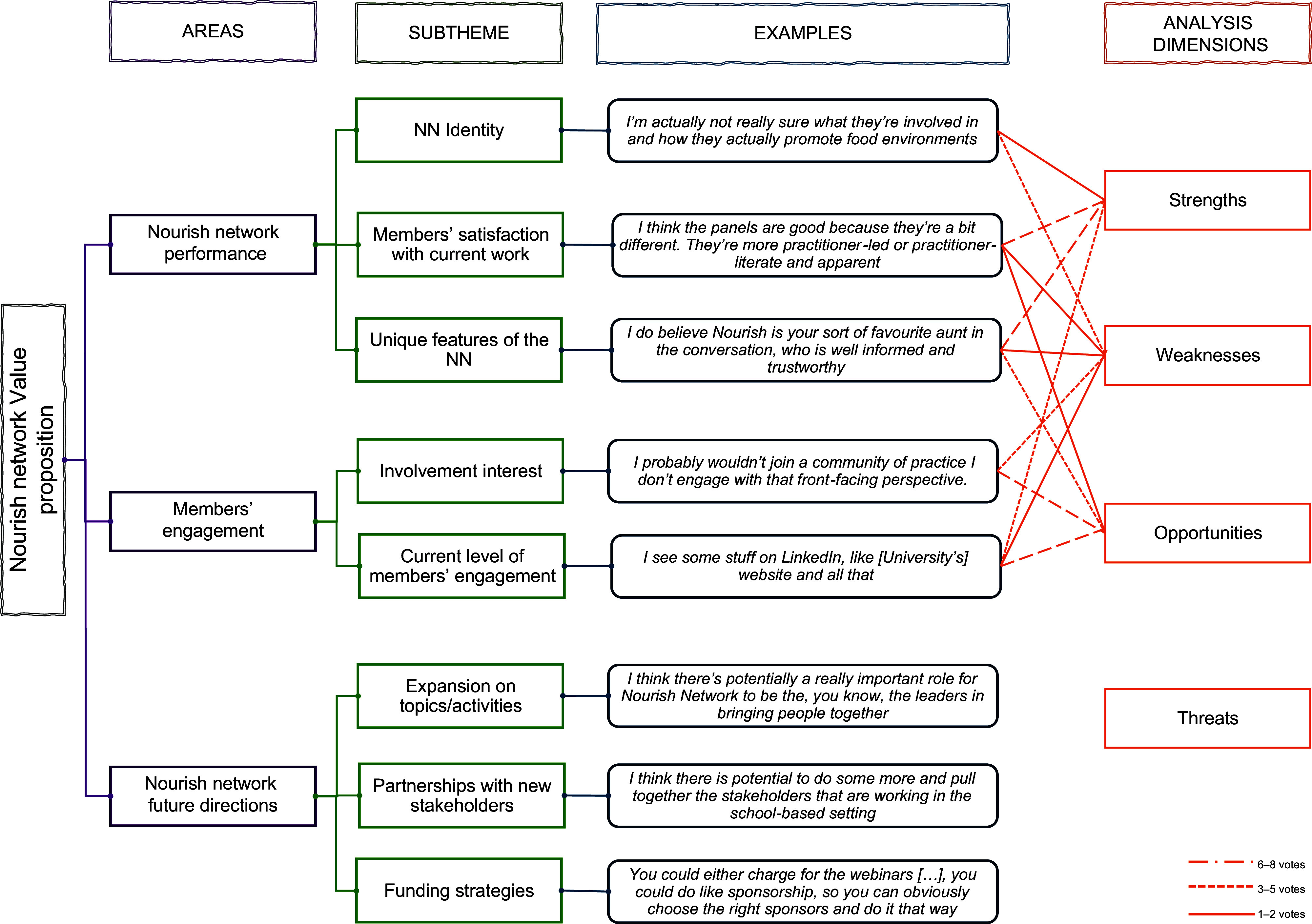
Summary of the data analysis across the interviews, surveys and the focus group.

Participant experiences and perspectives were organised into three major thematic areas (with eight minor themes also identified): (i) Nourish Network performance; (ii) Members’ engagement with the Nourish Network activities and (iii) Nourish Network future directions (Figure 1). Each theme is described in more detail below.

#### Nourish Network performance

Participants discussed their views on the NN’s overall performance and management processes since 2018. Three minor themes were identified:

##### Nourish Network identity

While some members were unclear of the full extent of the NN activities and goals, others demonstrated an understanding that the NN was involved in promoting healthy food environments, implementing policies to enhance the availability of healthy food and drinks and collaborating with various stakeholders, including local governments.

*I guess what I understood the Nourish Network to be doing is to assist in all of those different settings where people access food and drink to either help implement policy, or just make some own in-house policies to increase the supply of healthy food and drinks and work with the retailers and other people working in those settings to do that more efficiently and effectively (P-2).*

There was often uncertainty about the exact role of the NN and its connection to other organisations such as the university. Members considered that more clarity on which stakeholders were involved with the NN, tangible results outlining the NN performance and successes of the NN partnerships would foster transparency and accountability in the NN’s identity.

*[…] I must admit, in terms of what the Nourish Network does and what [the university] does […], it can get really confusing. in terms of the exact role of the Nourish Network. I’m not entirely sure … What the Nourish Network is, what it aims to do, and what its purpose is. Is it just to connect people who are interested and keep each other informed? Or is it to try and pull together people to come up with, you know, joint grant applications? Or is it for teaching purposes, I don’t know (P-3).*

##### Participants satisfaction with current work

Overall, members considered the NN to be a valuable source of connection and information. For example, some members considered the space it has provided to exchange healthy food retail ideas and collaboration among professionals from various backgrounds, including health, academia and community services, as the NN’s most valuable function. Additionally, the NN’s work was thought to be influential in addressing major food systems issues such as food (in)security by ensuring it was front of mind for diverse stakeholders making food retail decisions.

*The Nourish Network was the place that we seem to fit as a community, a key community stakeholder, but also a real hands-on real-time case study of what a food systems approach to nutrition is (P-4).*

##### Unique features

The NN was acknowledged for its unique focus on promoting healthy food and drinks by working with different settings to implement policies effectively. Some members also appreciated the diverse range of content that the NN shared on platforms like LinkedIn and their newsletters, as well as via webinars and forums. This trustworthy source of information was valued by participants as well as the opportunity the NN provided for input and learning without requiring payment.

*It’s hard for me to work out who my people are. I feel like Nourish is my people because they connect me with great data. But it is still very academic in its origins, and that’s a really important foundation (P-7).*

#### Members’ engagement with the Nourish Network activities

Participants discussed the way they engaged and would like to engage in the future with the NN’s activities. Two minor themes were identified:

##### Involvement and interest

Some members discussed their collaborations with the NN for three or four years, with these members commonly being the ones who understood how their work was closely aligned with the NN activities. Newer members mentioned that their possible lack of active collaboration might be related to lesser clarity on how to contribute effectively.

*But I would love to be able to [be more involved] […] being able to incorporate some of the work I do, you know, with the Nourish Network, all of that nutrition stuff to be more centred (P-6).*

##### Current level of members’ engagement

Overall, there was somewhat limited interest from members to be actively involved or volunteer to maintain the NN legacy. Lack of time, unaligned competing priorities in their workplaces, lack of funding for projects or collaborations with the NN and previously unpleasant experiences were some of the reasons hindering members’ active involvement.

*I currently sit in the periphery as there is no accountability to contribute more; perhaps more clarity around how I could specifically contribute would be helpful (Survey Participant).*

Despite some participants’ hesitance to get actively involved and the NN’s sharing of trustworthy and useful information, members preferred to engage more passively in nature through meetings, newsletters, websites and social media interactions. This was because of various reasons, such as time constraints to get involved in projects on top of workloads, access to specific content, reviewing information at their own pace or not feeling that their input could be helpful.

*From my perspective, I probably wouldn’t join a community of practice I don’t engage with, I guess engagement, that front-facing perspective. But certainly, I would recommend [colleges with more knowledge], and probably have more value added than myself (P7)*

#### Nourish Network future directions

Participants discussed their views on how the NN can move forward and improve its impact. Three minor themes were identified:

##### Expansion on topics or activities

Members indicated that they wanted the NN to further incorporate sustainability considerations into their work and the need for broader discussions of how healthy food retail changes interacted with broader food systems. For example, members discussed the NN’s potential to bring together diverse stakeholders and drive collective advocacy efforts to help promote healthy food retail, linking this work with climate change and sustainable food systems advocacy, actions and outcomes. Moreover, members suggested that they wanted the NN to also consider the importance of engaging with local governments to directly inform policy priorities and actions. Other relevant activities perceived to be valuable for NN members included bridging the gap between academia and practice in healthy food retail partnerships by helping health promotion practitioners to design or evaluate programs. Members further discussed how the NN could leverage their social media influence to support more widespread promotion of healthy food retail initiatives.

*[.] if the network, for example, was able to even help with more sort of program evaluation to show what’s working. If the network was able to do more stuff around data gathering, even literature reviews that kind of thing like bringing pieces of evidence together around problems. Those sorts of things would be really good from a practitioner and policy perspective (P-5)*

##### Partnerships with new stakeholders

To support partnerships among its membership, some participants suggested that the NN could assist in supporting collaborations between stakeholders, facilitating partnership funding opportunities and providing research and implementation support to improve food retail policy and interventions in diverse settings (i.e. schools, local governments, etc.). There was also interest in facilitating cross-state collaborations to learn from healthy food retail resources or methods used in other regions like Victoria or Western Australia. Other relevant stakeholders that members thought the NN could strengthen engagement with to diversify the network were the local community, technology experts, health professionals, dietitians, nutritionists, schools, the business sector and farmers.

*[…] the kind of benefit I think of being a member of a network is that first of all, you can be collegial and help each other, or, you know, inform each other, or give each other resources or methods, and share things […] collaborations, especially across states like, I think, that we’ve got a lot to learn from Victoria and perhaps Western Australia (P-3).*

##### Funding strategies

Finally, members suggested diverse funding options for the NN to sustain its activities, such as charging a fee for webinar attendance, seeking sponsorship, group memberships or consultancy work for community health services (i.e. evaluating programs or literature reviews) and obtaining more funding from university campus food and beverage offering services.

*You could either charge for the webinars, but if you don’t want to charge members for the Webinar, you could do like sponsorship, so you can obviously choose the right sponsors and do it that way (P-6).*

### Nourish Network Advisory Committee SWOT analysis

One focus group with nine members from the NNAC was conducted to further refine the future directions of the NN, using a SWOT analysis to identify interview and survey findings on NN performance and engagement. Figure 1 shows a summary of the data analysis and integration across the interviews, surveys and the NNAC focus group.

The NNAC considered members’ satisfaction with current work and the unique features of the NN as the greatest strengths of the NN. The weaknesses identified were not seen as significant, receiving only 6–8 votes. They included an unclear identity for the group and low member interest in active participation. However, these issues also present opportunities to enhance member engagement. Finally, the NNAC did not consider any threat preventing the NN to continue its activities. The NNAC elaborated on other strengths, weaknesses, opportunities or threats, which are summarised below:

#### Strengths

The NNAC considered that the NN’s main strength related to how it prioritised engagement with members from diverse organisations. Ensuring that all members were from a range of backgrounds and experiences was thought to have increased the NN credibility and trust among diverse stakeholder groups. Focus group participants highlighted the value and attention given to growing connections between members to ensure the NN serves as a hub that connects people with similar interests and goals. Moreover, the NNAC discussed leveraging collective years of experience through grant submissions from its multidisciplinary members to drive low-risk projects that can promote healthy food retail across sectors in the long term.

While some competitor networks were identified, NN was thought to give diverse stakeholders the ability to attend webinars and provide an open platform for knowledge sharing and collaboration. The NNAC suggested that this open-access platform fostered innovation and the widespread dissemination of information across the healthy food retail community (i.e. researchers, practitioners, retailers and policymakers). The NNAC identified the ongoing NN commitment to research translation has helped connect the sector with the latest research, particularly in the area of healthy food retail.

#### Weaknesses

The need for a clearer articulation of the NN’s mission and objectives was considered the main weakness of the network, as it could better convey the value of the NN to potential stakeholders and supporters. The NNAC further discussed the difficulty it has encountered engaging stakeholders in leadership positions. A suggested reason for the low level of participation, especially among team leads and key stakeholders – such as those that could provide resources, knowledge or funding – could be a lack of interest or a difference in priorities among team members. Additionally, there may be issues with accountability in completing tasks related to the NN. By enhancing communication within the working teams and strengthening leadership, it might be possible to engage these key stakeholders more effectively and encourage them to contribute towards achieving the NN goals.

#### Opportunities

The NNAC identified the opportunity for the NN to become a key resource hub for external consultancy and provide valuable knowledge and expertise to stakeholders working in healthy food retail. One avenue to support this consultancy idea was by leveraging its relationship with Deakin University to get support from PhD candidates working in the same space as the NN on specific tasks (e.g. systematic review). Finally, the NNAC identified that the NN could be expanded nationally by connecting with other healthy food retail outlets or prevention networks being built around the country to share best practices. This broader reach was perceived to help attract more stakeholders, strengthen collaborations and open up potential funding avenues and partnerships that may be limited on a smaller scale.

#### Threats

The NNAC identified that the most relevant threat to the NN was the proliferation of numerous nutrition-related, public health and sustainability networks across the country. It was indicated that as more stakeholders commence direct collaboration with food enterprises and businesses, competition from organisations with comparable offerings may intensify, leading to funding competition. Additionally, there was a noticeable decrease in the health promotion workforce in Victoria, Australia, with such shifts providing a risk in the labour market and potentially reduced interest in healthy food retail. A major internal challenge threatening the sustainability of the NN was discussed in relation to the lack of sufficient core funding to support its ongoing function and connectivity.

#### Recommendations

**Figure 2.**
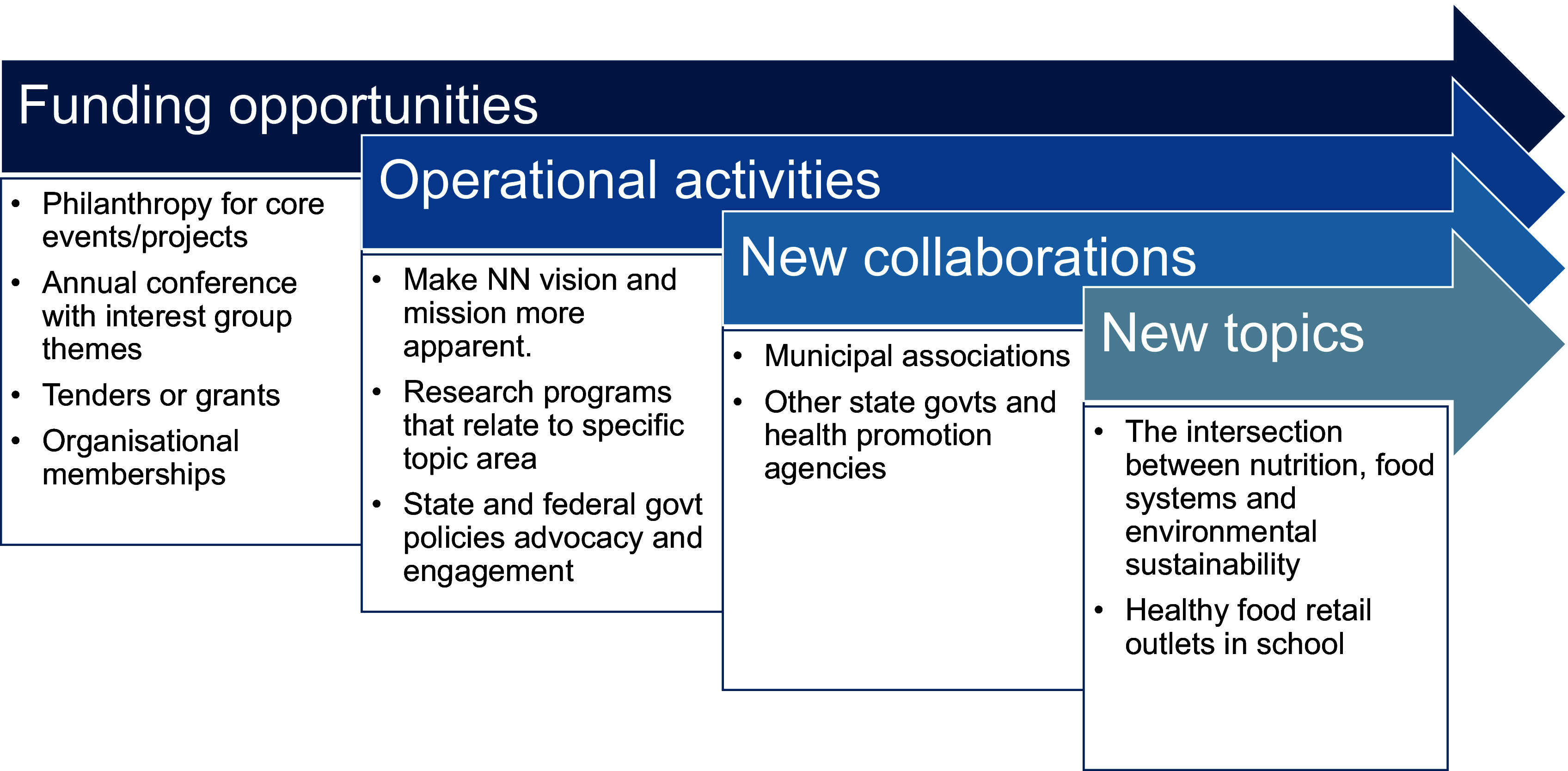
Summary of main recommendations to improve the Nourish Network’s performance and cross-sectoral engagement in healthy food retail.

To enhance the performance and cross-sectoral engagement of organisations like the NN in addressing complex social and health problems through collective impact^(9)^, several key recommendations can be implemented. First, operational activities should focus on stating clear, mutually reinforcing tasks where members leverage their unique strengths while ensuring alignment with current policy to keep the network updated. Revising and adjusting network goals as new collaborations emerge will help expand networks and explore topics relevant to the community’s needs. Additionally, implementing agreed measurement systems will ensure accountability and alignment across participating organisations, while continuous communication will promote trust and recognition of collective efforts. Although funding is not a central tenet of collective impact, the backbone organisation and all members must ensure the financial sustainability of the network. Figure 2 portrays the specific recommendations that the NN can consider.

## Discussion

This study assessed the current state of the NN through the views and opinions of its members. Our study provides insights into the functioning, strengths and challenges of an Australian healthy food retail network (NN), with implications that extend beyond the Australian context, as collaborative networks are becoming increasingly central to public health strategies globally. Understanding how such networks operate, engage members and sustain momentum is critical^(18,30)^. The SWOT analysis expanded on members’ views to identify intervention points that can be leveraged in future planning to sustain the NN’s activities. Such participatory evaluation methods are increasingly recognised as essential for adaptive and context-sensitive public health interventions^(7)^.

Evaluations of collaborative networks have employed a variety of frameworks to assess structure, function and impact. For example, clinical networks have often been evaluated using mixed-method approaches that combine social network analysis with qualitative assessments of governance and member engagement^(31)^. Similarly, coalitions and public health networks have been assessed through models such as the Community Coalition Action Theory and Collective Impact, which emphasise shared goals, continuous communication and backbone support^(17)^. These approaches highlight the importance of both relational and structural dimensions in determining network effectiveness. The current study builds on this literature by applying a participatory SWOT analysis, which expanded on members’ views to identify internal dynamics and strategic opportunities of the NN. By integrating member perspectives and reflexive analysis, this study contributes a context-sensitive model for evaluating networks that operate in complex, multi-sectoral environments.

Our results suggest a somewhat unclear understanding of the vision and mission of the NN among participants, which may limit stakeholder engagement and the level of involvement in NN activities. This issue echoes findings from international evaluations of public health networks, where that ambiguity in purpose can hinder member engagement and dilute collective impact^(32,33)^. Moreover, the collective impact literature emphasises the need for a common agenda and shared measurement systems as foundational to effective collaboration^(11,17)^. This literature further highlights the importance of effective communication strategies and participatory governance structures that promote a shared understanding and ownership.

Literature on research networks has acknowledged that network members should be aware of the network’s structure and features, attend to the network’s function and dedicate time to enhancing its effectiveness^(34)^. Our results showed that there is an opportunity for the NN to strengthen internal processes to clarify membership understanding of its purpose and values. This lack of clarity may be due to the broad scope of NN, which can lead to members in one area not fully understanding the key points of other elements of the network that are unrelated to their specific area. A network’s effectiveness could be influenced by how its members interact and behave^(35,36)^. While being broad has both advantages and disadvantages, a more focused approach could enhance stakeholder investment and engagement, making it easier for everyone to grasp the overarching goals of the NN. Previous literature has shown that aligning actions and priorities across networks can be challenging; however, having a clear view of the desired outputs and achievements can improve the connectivity and collaboration of network members^(31,37)^. To establish a clear and compelling purpose, the NN or any similar network could identify unique and valuable opportunities to promote its core mission and continually revisit this purpose as the network grows and evolves.

While the NN’s purpose was unclear for its members, participants valued the trustworthiness of the healthy food retail information commonly provided through various channels (i.e. webinars, social media or newsletters) and the NN’s ability to connect stakeholders across the food system and foster collaboration. The NN’s emphasis on trust, inclusivity and knowledge sharing aligns with principles of effective network and community building^(18)^, demonstrating that even in resource-constrained settings, fostering a sense of belonging and mutual support can enhance network resilience and perceived value^(38,39)^. These relational assets are foundational for networks aiming to address complex, system-level challenges such as food insecurity^(38)^. The NN members’ preference for passive engagement methods (e.g. newsletters and webinars) over active participation is not aligned with the literature that suggests active collaboration is a universal marker of network success^(18)^. This nuance highlights the need for flexible engagement strategies that cater to the diverse capacities and motivations of members.

The NN was established within an academic institution, which provided the network management, support and mechanisms to bring diverse stakeholders together. While the NN is not identified as either a clinical or research network, it exhibits characteristics of the ’enclave’ type of network as described by Goodwin *et al.*^(40)^, featuring a non-hierarchical organisation similar to some government-funded clinical networks^(31)^. This type of structure enables dynamic and collaborative decision-making processes, ensuring effective communication and coordination among members^(40)^. However, unlike clinical networks, funding was not provided to the NN by its members, institutions or the government, and a lack of resources creates tensions in prioritising academic outputs or return on investment as primary outcome measures in evaluations^(34)^. Networks like the NN can also find it challenging to measure their impact and sustain their activities, as there is often a delay related to the benefits of their actions^(41)^. Evidence suggests that securing long-term financial support can also be a challenge for networks that focus on establishing and holding partnerships^(37)^. To ensure long-term impact, the NN and similar networks must consider sustainability strategies that include and go beyond funding. These include cultivating leadership across sectors, embedding evaluation into routine practice and leveraging policy windows to institutionalise successful practices^(33,42)^.

Another key benefit of networks is the crucial role they play in staying current with emerging research topics, identifying knowledge gaps and developing innovative technologies^(37)^. This proactive approach to knowledge sharing directly supports the overarching objective of enhancing research capacity, ensuring that diverse stakeholders have access to the latest advancements and methodologies in their respective fields^(43,44)^. Our results suggest that the practical strategies taken by the NN, such as forming working groups, hosting workshops and organising discussion panels, are essential for building engagement and collaboration among stakeholders on timely topics. This is particularly relevant for governments and health authorities interested in promoting health-enabling food environments, whereby strengthening research capacity has been shown to lead to more effective public health initiatives that result in short and long-term improvements in community health outcomes^(19)^.

The strengths of our study include the comprehensive set of data analysis techniques employed, which provided an in-depth understanding of NN members’ experiences and identified practical leverage points for improvement. Our analytical model can be adopted in regular assessments and incorporated into the monitoring of similar healthy food retail networks. Additionally, the use of a SWOT-informed participatory approach strengthens the credibility of the analysis and empowers members to co-construct the network’s future direction. A limitation of this study is that only sixteen NN members (eight NNAC and eight NN members) agreed to participate. This limited number of participants may not capture the full diversity of perspectives or experiences within the broader NN membership, potentially leading to a limited understanding of future actions that the NN could take. Moreover, the sample may have introduced further bias (e.g. availability, desirability to participate or engagement level and time) or limited the identification of insights into how members from different sectors or with different engagement levels experienced collaboration within the NN. To address this, future research could incorporate comparative or mixed-method approaches from the beginning of these types of initiatives to better illuminate multi-disciplinary perspectives and engagement dynamics. Finally, to our knowledge, the NN is the first nutrition and food network of its kind to have been rigorously evaluated, thereby limiting comparisons with other types of networks.

### Conclusion

For the NN and similar networks to effectively drive healthy food retail changes across sectors, a clear and updated vision, along with ongoing efforts to strengthen engagement among those interested in promoting health-enabling food environments, is necessary. This can include consolidating its unique set of activities of knowledge sharing and cultivating meaningful relationships and partnerships, but it will also require funding support. Sustaining the NN’s efforts may support the development of research in areas that require additional resources and infrastructure. Future research could leverage the NN’s focus on measurable outputs by fostering more structured collaborations and partnerships.

## Supporting information

Vargas et al. supplementary material Vargas et al. supplementary material

## Supplementary material

For supplementary material accompanying this paper visit https://doi.org/10.1017/S1368980025100992
