# Supplementary material for: Evaluating the Nourish Network: a multi-sector collective initiative to progress healthy food retail in Australia
Source: Public Health Nutr. 2025 Sep 4;28(1):e155. doi: 10.1017/S1368980025100992 (PMC12516627; doi:10.1017/S1368980025100992)
Supplement: Vargas et al. supplementary material [file S1368980025100992sup001.docx]

Table S1. Interview guide

| **Opening/Rapport** | |
| --- | --- |
| **Topic** | **Content** |
| Self-introduction | Name, background |
| Plain language explanation | To identify products and/or service opportunities for the Nourish Network (NN) to continue supporting its members. |
| Record consent for participation | Verbal consent from the participant to be recorded and reiteration of the use of information in a non-identifiable form |
| Participant introduction | Understanding the participant's positioning regarding the research topic |
| **Main interview** | |
| **Topic** | **Type of questions** |
| Opening questions | - How long have you been part of the NN? - How do you normally engage with the NN? (e.g., seminars, interest groups) - Would you like to be involved differently (more/less)? – How? |
| Structure and governance. | 1. Please tell us what you know about Nourish Network.    1. What do you think Nourish Network does?       1. And what do you think it aims to do?    2. What is your role in your org?    3. How did you come to know about Nourish Network?    4. What is the value of NN (in general/their org/sector/community?       1. Alignment NN and their aims?       2. Organisation, community...    5. What are the strengths of the NN? What are the limitations? |
|  | 1. Please describe the way in which you or your organisation are interested or involved in the healthy food retail space.    1. What is their overall organisational goal?    2. Challenges with accessing NN    3. Facilitators and opportunities with accessing NN |
| Impact of the NN | 1. What are the key problems or pain points that you face?    1. Do we need to be more specific here? E.g.: in relation to buying in, selling & promoting healthy food?    2. Internal to their work and achieving their mission about food?    3. If you could wave a magic wand to solve a work problem, what would that magic wand do?    4. Where do you feel you are stretched the thinnest in your work? What do you really wish you had more help with?    5. How can the NN help you to solve x, y z problem?    6. Is there an opportunity to connect people with resources/academics? Do you think that would help build credibility? how? 2. What should Nourish Network be doing that would help you achieve healthier food provision outcomes sooner?    1. For example: healthier options available from suppliers, government mandates, more toolkits/guides to inform etc    2. Support efforts to address any structural issues    3. Evidence based ideas and examples    4. Can you see something that you feel needs doing that you think Nourish Network would be perfect to help get done? 3. For example: a healthy food recognition award/program |
| Functioning and opportunities | 1. Do you think NN would benefit from more formal partnerships?    1. What other people or groups should we involve in the NN? E.g. Food Foundation (UK), VicHealth, Retail Traders Association, Caterer's Association (local, national and global).    2. Are we engaging with key decision makers that would support your work? why/why not? How can we do this better? 2. What questions should the NN be asking that we are not?    1. What can the NN do so people/organisations invest on us?    2. What services are you interested in obtaining from the NN? Internship, researcher, support?    3. What are the strengths of the NN that are valuable for participants for investment? |
| **Closing** | |
| **Topic** | **Rationale** |
| End o interview | Thank you for taking the time to participate in our research project. Your time and expertise are invaluable to us. |

Table S2 Nourish Network Advisory Committee Session

| **Date:** 26^th^ July 2024  **Time:** 2:30 - 4:00 pm | |
| --- | --- |
| **Topic** | **Content** |
| Aim: | To identify the Nourish Network advisory committee responses to members’ views. |
| Objectives: | 1. Report to the advisory committee on Nourish Network members' views to continue supporting them. 2. Classify those views as strengths, weaknesses, opportunities and threats (SWOT) to inform the Nourish Network’s future planning. 3. Gain input from the advisory committee on Nourish Network’s future |
| Session agenda | \| - Welcome - Acknowledge to country - Ethics review and consent [recording starting] - Session aim and objectives - Presentation of the interviews and survey analysis - Input and reflections \| 2:30 - 2:35  2:35 – 2:40  2:40 – 2:45  2:45 – 2:48  2:48 – 3:20  3:20 – 3:30 \| \| --- \| --- \| |
